# Subcutaneous dirofilariasis in a 25-year-old male patient in Belgium on ultrasonography: a case report

**DOI:** 10.1186/s13256-023-04242-z

**Published:** 2024-01-14

**Authors:** Scott Klerkx, Caroline Venstermans

**Affiliations:** Department of Imaging & Pathology, GZA Hospitals Sint-Augustinus, Oosterveldlaan 24, 2610 Wilrijk, Antwerp Belgium

**Keywords:** Subcutaneous dirofilariasis, *Dirofilaria repens*, Ultrasound, Case report

## Abstract

**Background:**

Subcutaneous dirofilariasis is a parasitic zoonosis commonly described in Canidae but rarely seen in humans. Most physicians are unfamiliar with this disease, especially in nonendemic areas, which can lead to medication error and diagnostic and treatment delay. To the best of our knowledge, no previous case of subcutaneous dirofilariasis preoperatively diagnosed on ultrasound has been described in Western Europe.

**Case presentation:**

A 25-year-old Belgian male patient presented with a subcutaneous nodule in the epigastric region. Ultrasound investigation showed a typical cystic lesion with an internal serpiginous structure with echogenic lines, and there was active twirling movement of this serpentine structure during investigation, pathognomonic for subcutaneous dirofilariasis. Surgical extirpation was performed, and the diagnosis was histopathologically confirmed.

**Conclusion:**

Subcutaneous dirofilariasis has a characteristic appearance on ultrasound but is not well known in nonendemic areas, often leading to diagnostic delay and initial incorrect treatment. More knowledge of this disease and of its characteristic ultrasound appearance will hopefully lead to better patient care.

## Introduction

Dirofilariasis is a parasitic zoonosis caused by nematodes of the genus *Dirofilaria*. There are over 20 dirofiliaris species, which mostly infect dogs, cats, and wild carnivores and are being transmitted by multiple mosquito species [1]. In these mosquito vectors, microfilariae mature into infectious larvae after which transmission takes place when the mosquito takes a blood meal. Canidae are the main reservoir but incidentally the nematodes can be transmitted to humans. In human beings, complete sexual maturation of the microfilaria cannot occur due to the host defense, preventing the expression of larvae in the blood stream. Therefore, no further transmission to other hosts takes place [2]. Most cases have been described of the species *Dirofilaria repens* and *Dirofilaria immitis* regarding human infections [3]. *D. immitis* causes deep organ infections, mostly in the pulmonary arteries and right ventricle of the heart, and *D. repens* mainly causes subcutaneous and ocular infection [3].

Most human infections by *D. repens* occur in the Mediterranean region and other subtropic and tropic places in Europe, Asia, and Africa [2, 3]. Sri Lanka is the most afflicted country in Asia, where 30–60% of the dog population is infected with *D. repens* in some parts of the country [4]. The most reported symptoms of subcutaneous dirofilariasis are migrating subcutaneous lesions, intermittent painful erythema, and itching [5]. Subcutaneous dirofilariasis should be treated with complete surgical extirpation of the lesion. Antihelminthic drugs are usually only required when immunodeficiency is present, since a normal functioning immune system is able to prevent reproduction and transmission [2, 5].

On ultrasound, subcutaneous dirofilariasis presents as a hypoechoic nodular lesion with an internal serpiginous structure with internal parallel echogenic walls and an anechoic center. Sometimes active motion of microfilariae is visible, similar to the “filarial dance sign” seen in lymphatic filariasis caused by filarial nematodes of the species *Wuchereria bancrofti*, *Brugia malayi*, and *Brugia timori* [6].

Human subcutaneous dirofilariasis by *D. repens* is scarcely reported in nonendemic areas such as Belgium, and in almost all cases the diagnosis is made after excisional biopsy, often with initial misdiagnosis, significant treatment delay, and medication error.

To the best of our knowledge, this case is the first well-reported human subcutaneous dirofilariasis preoperatively diagnosed on ultrasound in Western Europe.

## Case presentation

**Fig. 1:**
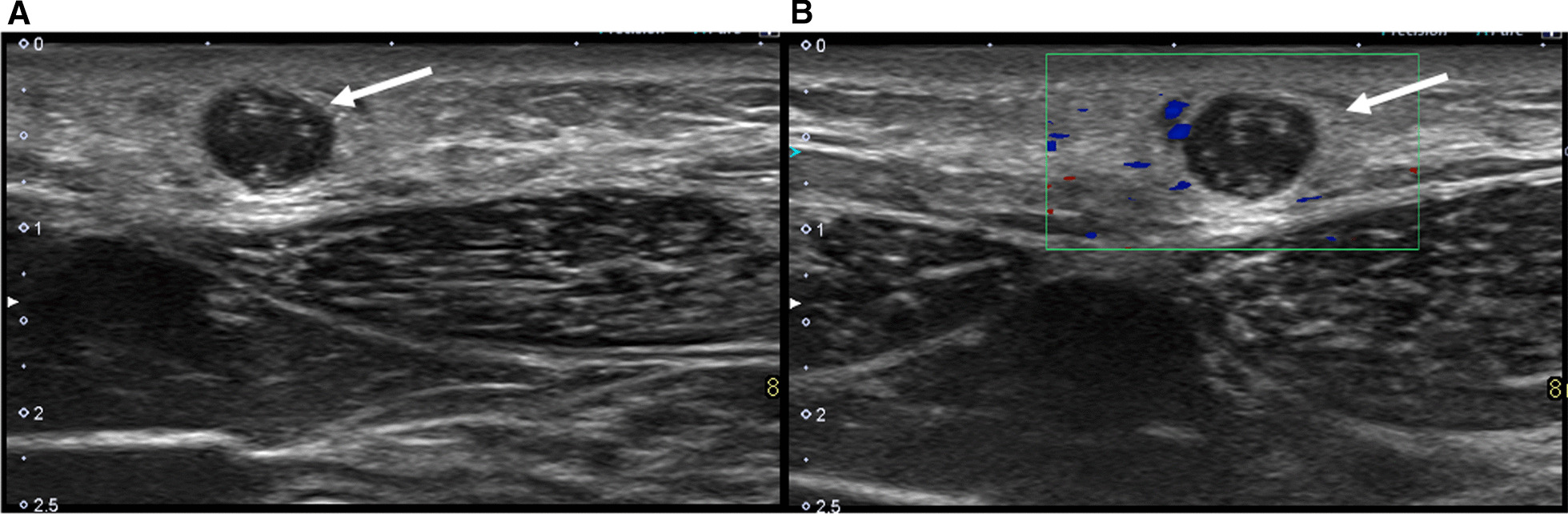
**A** Ultrasound shows a cystic structure with a diameter of circa 8 mm and an internal tubular serpiginous structure with parallel echogenic lines (arrow). During the investigation, there was active twirling movement of the serpentine structure. **B** Doppler ultrasonography showed no significant vascularization of the lesion

**Fig. 2:**
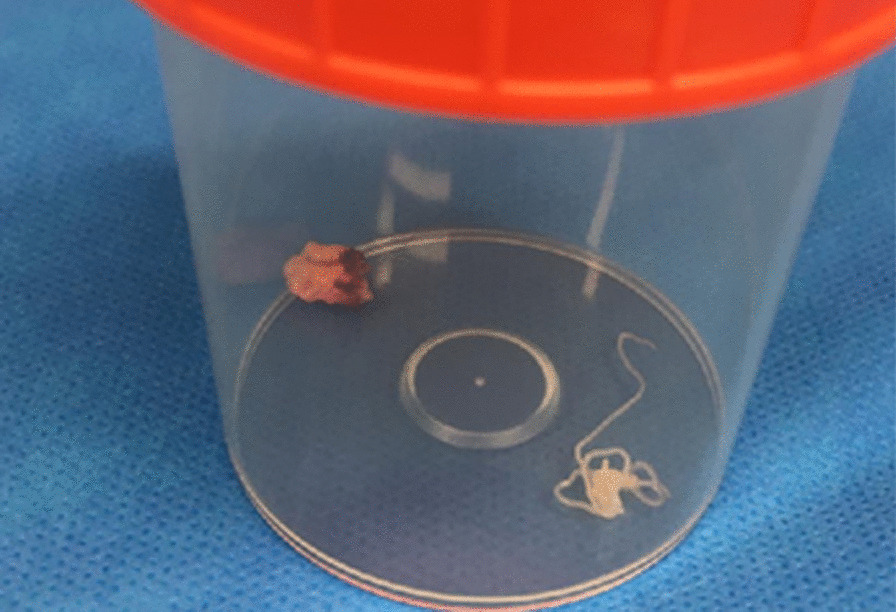
The excisional biopsy specimen contains a lobulated subcutaneous mass on the left side and the worm on the right side, which was later histopathologically confirmed as *Dirofilaria repens*

A 25-year-old native male patient from Flanders (Belgium) with no relevant medical history presented to the general practitioner (GP) with an infrasternal, clinically palpable nodule in the epigastric region with localized irritation and itching. A cyst or benign lymphadenopathy was suspected and initially no treatment was started but at the patient’s request, an ultrasound examination was conducted to exclude other pathology. Approximately 6 months before symptom onset, the patient had been in France and central Italy. The leukocyte count and eosinophil count was within normal limits. Symptoms started about 2 weeks before ultrasonography. On ultrasound, there was a cystic structure with a diameter of circa 8 mm with an internal tubular serpiginous structure with parallel echogenic lines (Fig. 1A). During the investigation, there was active twirling movement of the serpentine structure, similar to the “filarial dance sign” seen in lymphatic filariasis. The lesion showed no vascularization during Doppler investigation, and there was no penetration through the fascia with an intact appearance of the underlying musculature (Fig. 1B). The preoperative diagnosis of a subcutaneous dirofilariasis was made and surgical extirpation was performed (Fig. 2). The excisional specimen was send to the Institute of Tropical Medicine, where histopathological assessment confirmed a subcutaneous dirofilariasis caused by *D. repens*.

## Discussion

Human subcutaneous dirofilariasis is a zoonosis caused by infection due to nematodes of the genus *Dirofilaria*, with the majority caused by *D. repens*. They are transmitted by mosquito vectors and Canidae are the main reservoir. Incidentally, transmission to humans are dead ends in *Dirofilaria* infestation due to the fact that the worms do not attain maturity and are unable to express microfilaria in the blood stream. Therefore, serological tests are usually not useful and systemic therapy is not indicated. Eosinophilia is an inconsistent finding depending on immune response [7]. Subcutaneous dirofilariasis should be considered if a patient presents with a migrating subcutaneous nodule, also in nonendemic areas.

Ultrasonography is the first-line imaging technique with high specificity showing a cystic nodule with an internal serpiginous structure consisting of parallel echogenic lines. Active twirling movement of the serpiginous structure can be seen, as described in lymphatic filariasis as the “filarial dance sign.” Magetic resonance imaging (MRI) can rarely be valuable if extension to the muscles or joints is expected [7, 8].

The subcutaneous lesion should be treated with total surgical extirpation of the lesion. Anthelmintic treatment (e.g., albendazole) is not recommended in most cases but can be useful for immunocompromised patients or migratory lesions, especially in the face, because these drugs promote fixation, after which the lesion can be surgically removed [2, 5].

## Conclusion

Despite the characteristic imaging features of subcutaneous dirofilariasis on ultrasound, these lesions are usually removed surgically with significant delay and without preoperative ultrasonographic investigation, especially in nonendemic areas. Early and correct diagnosis prevents significant patient distress and prevents medication error (e.g., inappropriate antibiotic use).

Hopefully, this case report will create more knowledge of the characteristic ultrasound appearance of subcutaneous dirofilariasis and more awareness of the disease in general, leading to better patient care with early and correct diagnosis and treatment.

## Data Availability

All data analyzed during this study are included in this published article.
